# Inflation using hydrogen improves donor lung quality by regulating mitochondrial function during cold ischemia phase

**DOI:** 10.1186/s12890-023-02504-6

**Published:** 2023-06-17

**Authors:** Le Duan, Lini Quan, Bin Zheng, Zhe Li, Guangchao Zhang, Mengdi Zhang, Huacheng Zhou

**Affiliations:** 1 Department of Anesthesiology, the Second Affiliated Hospital of Harbin Medical University, Harbin, China; 2 Department of Pain Medicine, the Fourth Affiliated Hospital of Harbin Medical University, No.37, Yiyuan Street, Nangang District, 150001 Harbin, China; 3 Department of Anesthesiology, Jinan Central Hospital, Cheeloo College of Medicine, Shandong University, Jinan, China; 4 Department of Anesthesiology, Second Affiliated Hospital of Xi’an Jiaotong University, Xi’an, China; 5 Department of Anesthesiology, Affiliated Hospital of Nantong University, Nantong, China; 6 Department of Anesthesiology, the Fourth Affiliated Hospital of Harbin Medical University, No.37, Yiyuan Street, Nangang District, 150001 Harbin, China

**Keywords:** Hydrogen, Lung transplantation, Mitochondrial function, Donor lung quality, Cold ischemia, Inflammation

## Abstract

**Background:**

Mitochondrial dysfunction results in poor organ quality, negatively affecting the outcomes of lung transplantation. Whether hydrogen benefits mitochondrial function in cold-preserved donors remain unclear. The present study assessed the effect of hydrogen on mitochondrial dysfunction in donor lung injury during cold ischemia phase (CIP) and explored the underlying regulatory mechanism.

**Methods:**

Left donor lungs were inflated using 40% oxygen + 60% nitrogen (O group), or 3% hydrogen + 40% oxygen + 57% nitrogen (H group). Donor lungs were deflated in the control group and were harvested immediately after perfusion in the sham group (*n* = 10). Inflammation, oxidative stress, apoptosis, histological changes, mitochondrial energy metabolism, and mitochondrial structure and function were assessed. The expression of nuclear factor erythroid 2-related factor 2 (Nrf2) and heme oxygenase-1 (HO-1) were also analyzed.

**Results:**

Compared with the sham group, inflammatory response, oxidative stress, histopathological changes, and mitochondrial damage were severe in the other three groups. However, these injury indexes were remarkably decreased in O and H groups, with increased Nrf2 and HO-1 levels, elevated mitochondrial biosynthesis, inhibition of anaerobic glycolysis and restored mitochondrial structure and function compared with the control group. Moreover, inflation using hydrogen contributed to stronger protection against mitochondrial dysfunction and higher levels of Nrf2 and HO-1 when comparing with O group.

**Conclusions:**

Lung inflation using hydrogen during CIP may improve donor lung quality by mitigating mitochondrial structural anomalies, enhancing mitochondrial function, and alleviating oxidative stress, inflammation, and apoptosis, which may be achieved through activation of the Nrf2/HO-1 pathway.

**Supplementary Information:**

The online version contains supplementary material available at 10.1186/s12890-023-02504-6.

## Background

Lung transplantation (LTx) is currently the most effective treatment for end-stage pulmonary diseases, with almost 70,000 adult LTx procedures registered worldwide per year [1]. However, donor lung contusion(s), infection(s), neurogenic pulmonary edema, and ischemic injury contribute to poor donor lung quality, leading to a suitable utilization rate of < 20% [2, 3]. To reduce mortality among LTx candidates on wait lists, many measures are required to expand the donor pool and improve donor quality, such as broadening the use of organs, enhancing donor authorization, rehabilitating poor quality organs, and optimizing donor management [4]. Therefore, optimizing donor lung quality remains a noteworthy focus in improving recipient prognosis.

Among donor lung preservation methods, static cold storage remains the gold-standard strategy worldwide, with the advantages of simplicity, convenience, and low cost-effectiveness compared with machine perfusion [5]. Although static cold storage can decrease metabolic rate, maintain transmembrane electrochemical gradients, and suspend the activation of apoptotic biochemical pathways, lung deflation still causes damage to alveolar cells and alveolocapillary membranes, inducing mechanical injury owing to shear stress(es) during re-expansion [6]. Additionally, hypothermia itself abolishes the activation of the sodium pump, boosts intracellular calcium overload, and leads to bursts in the production of reactive oxygen species (ROS), resulting in graft function impairment. Consequently, lung inflation treatment can be an important strategy for promoting function after preservation by protecting the delicate balance between the beneficial and deleterious effects of hypothermic cooling, with adverse effects minimized and protective actions maximized at approximately 4 ℃ [5].

Owing to its low molecular weight, hydrogen (H_2_) is a therapeutic antioxidant via selective scavenging of cytotoxic oxygen radicals [7]. It can directly permeate lung tissue through external respiration and spread into the cytoplasm, mitochondria and nuclei. Haam et al*.* reported that 2% H_2_ inhalation could improve porcine lung graft function during ex vivo lung perfusion (EVLP), via inhibition of oxidative stress and inflammation [8]. Similarly, we found that lung expansion using 3% H_2_ + 40% oxygen (O_2_) exerted a stronger protective effect than 40% O_2_ alone on alleviating ischemia–reperfusion injury (IRI) of lung graft during cold ischemia [9, 10]. In addition, we also determined that 3% H_2_ mitigated structural distortion of the mitochondria by anti-inflammation in endothelial cells during the cold ischemia phase (CIP) [11]. Although H_2_ exerts a protective effect on the donor lung, the regulatory mechanism remains to be clarified.

Mitochondrial dysfunction is closely related to many lung diseases or processes, such as coronavirus disease 2019 [12], lung IRI, fibrosis [13] and pulmonary edema [14], with manifestations of decreased mitochondrial membrane potential (MMP), abnormal energy metabolism, and redox imbalance. Mitochondrial function can be regulated by multiple signal pathways, one of which is nuclear factor erythroid 2-related factor 2 (Nrf2)/heme oxygenase-1 (HO-1). Under circumstances of oxidative stress, Nrf2 can translocate, activate downstream antioxidant enzymes (i.e., HO-1 and superoxide dismutase [SOD]) and impede ROS generation [15]. However, the effect of H_2_ on mitochondrial dysfunction during CIP is unclear. The purpose of the present study, therefore, was to investigate whether H_2_ has the capacity to protect against mitochondrial dysfunction and to explore the regulatory mechanism to improve donor lung quality during CIP.

## Methods

Pathogen-free, male Wistar rats (weight 250-300 g) were purchased from Harbin Medical University (Harbin, Heilongjiang, China). Based on the LTx model of our research team [9–11], the male rats were chosen in this study. The animals were housed in individual cages in a temperature-controlled room with a 12-h light/dark cycle, with ad libitum access to food and water. All animal experimental protocols were performed in accordance with the ARRIVE guidelines. All methods were carried out in accordance with relevant guidelines and regulations and approved by the Ethics Committee of Harbin Medical University.

### Experimental protocol

The rats were anesthetized using an intraperitoneal injection of pentobarbital sodium (60 mg/kg) and were intubated endotracheally through tracheotomy. The animals were ventilated with room air at a tidal volume of 10 ml/kg and a rate of 40–60 breaths/min (Model 683, Harvard Apparatus, MA, USA). Five minutes after intravenous administration of sodium heparin (200 U/kg), a median sternotomy was performed, and the lungs were flushed with 20 ml low-potassium dextran (LPD) solution (prepared by Harbin Medical University) via the pulmonary artery at a pressure of 20 cmH_2_O. The left lung was then harvested and stored at 4 ℃ for appropriate ventilation treatment.

All rats were randomly divided into four groups (*n* = 10 each). The left lungs in the sham group were harvested for analysis immediately after flushing, and were deflated in the control group and inflated with 40% O_2_ + 60% N_2_ in the oxygen (O) group, or 3% H_2_ + 40% O_2_ + 57% N_2_ in the hydrogen (H) group at 5 ml/kg bodyweight. Then the lungs were stored in 4 ℃ LPD for 4 h. The mixed gases were replaced every 20 min with a three-way pipe connected to an airtight injector during CIP. H_2_ concentration was monitored using a gas analyzer (S/N 32590; DATEX Ohmeda, Finland).

### Assessment of oxidative stress and inflammatory cytokines

Left superior pulmonary tissue was desiccated at 80 °C for 72 h to measure the wet-dry weight ratio (W/D); the inferior part was frozen at -80 ℃. Tissue was homogenized to determine malondialdehyde (MDA), myeloperoxidase (MPO) and SOD activity (Jiancheng BioTechnology, Nanjing, China). The levels of interleukin (IL)-6, IL-10, and tumor necrosis factor-alpha (TNF-α) were measured using a commercially available enzyme-linked immunosorbent assay kit (Jiancheng BioTechnology, Nanjing, China).

### Histological examination

The middle section of the left lung was fixed in 4% paraformaldehyde, embedded in paraffin, cut into Sects. (4 μm thick), and stained with hematoxylin and eosin. The lung injury score (LIS) was evaluated based on neutrophil infiltration, airway epithelial cell damage, interstitial edema, hemorrhage, and hyaline membrane formation, as follows: normal = 0; minimal change = 1; mild change = 2; moderate change = 3; and severe change = 4 [16]. All sections were evaluated using light microscopy by a pathologist who was blinded to the study design.

### Apoptosis assay

Apoptosis of alveolar epithelial cells was detected using a terminal deoxynucleotidyl transferase-mediated dUTP nick end labeling (TUNEL) assay (Roche, Boehringer Mannheim, Germany) and immunohistochemical staining of caspase-3 using a commercially available kit in accordance with manufacturer’s instructions (Cell Signaling Technology Inc, Boston, MA USA). The apoptotic index (AI) was calculated by counting the positive cells in the TUNEL assay [17], while immunohistochemical score (IHS) [18] was calculated by multiplying the quantity score (no staining = 0; 1%–10% of cells = 1; 11%–50% = 2; 51%–80% = 3; and 81%–100% = 4) and the staining intensity score (negative = 0; weak = 1; moderate = 2; and strong = 3) in five random high-power (× 40) fields.

### Measurement of energy metabolism and mitochondrial lipid peroxidation

The left inferior lungs were analyzed for lactic acid, glucose and pyruvic acid in accordance with a standardized protocol (Jiancheng BioTechnology, Nanjing, China). Adenosine triphosphate (ATP) content was measured using an ATP bioluminescent assay kit (Genmed Scientifics Inc., USA). Left middle sample was homogenized and centrifuged at 4℃. The supernatant was centrifuged again at 7000 × *g* for 10 min at 4℃. Crude mitochondrial preparations were collected according to the manufacturer’s protocol (Biovision Inc., CA, USA). Protein content was measured using a BCA protein assay kit (Beyotime Biotechnology, Shanghai, China). Finally, the supernatant was analyzed for mitochondrial MDA (mMDA), mitochondrial glutathione (mGSH) and mitochondrial glutathione disulfide (mGSSG) based on protocols provided by the manufacturer (Jiancheng BioTechnology, Nanjing, China).

### Observation of mitochondrial structure

Sections of lung grafts (1 mm^3^) were collected and fixed in 2.5% glutaraldehyde, embedded in epoxy resin, and cut into slices 40–50 nm thick. The ultrathin section was stained with uranyl acetate and lead citrate. Mitochondria in type II alveolar epithelial cells were examined using transmission electron microscopy (H-7650, Hitachi, Tokyo, Japan). The degree of mitochondrial injury was evaluated according to Flameng score, as follows: 0, normal mitochondrial structure, intact particles; 1, normal mitochondrial structure with missing particles; 2, swollen mitochondrial, transparent and clear matrix; 3, ruptured cristae, transparent mitochondrial matrix and condensed mitochondrial; and 4, ruptured mitochondrial inner and outer membrane, disrupted structure [16]. The samples were evaluated by a specialist who was blinded to the study design.

### Detection of MMP and mitochondrial swelling

MMP was analyzed in isolated mitochondria using a fluorescent probe JC-1 staining kit (Beyotime Biotechnology, Shanghai, China). JC-1 exists as an aggregated form (red fluorescence with excitation at 525 nm, emission at 590 nm) in the matrix of mitochondria with the normal MMP and converted into the monomeric form of collapsed MMP (green fluorescence with excitation at 490 nm, emission at 530 nm). MMP was calculated according to red/green fluorescence intensity. Mitochondrial swelling was assayed as the decrease in absorbance at 520 nm by using a commercially available assay kit in accordance with manufacturer’s instructions (Genmed Scientifics Inc., USA).

### Protein expression of Nrf2 and HO-1 determined by Western blot

Left lung tissue was finely homogenized in ice-cold radioimmunoprecipitation assay (i.e., “RIPA”) buffer. Total protein was extracted and measured using an enhanced BCA protein assay kit (Beyotime Biotechnology, Shanghai, China). The protein samples were separated by 10% sodium dodecyl sulfate–polyacrylamide gel electrophoresis and transferred to polyvinylidene fluoride membranes. The membranes were then blocked with 5% fat-free milk for 2 h, and incubated with primary antibodies against Nrf2 (1:1000, Wanleibio, Shenyang, China), HO-1 (1:10,000, Abcam, Cambridge, UK) and β-actin (1:10,000, Bioss, Beijing, China) at 4 ℃ for 24 h. After three washes, the membranes were incubated with secondary antibody (1:5000, Boster, Wuhan, China) for 1 h at room temperature. All blots were developed using ECL-Plus reagents and quantified using ImageJ software version 1.61 (NIH, Bethesda, MD, USA) and normalized to β-actin.

### Statistical analysis

Data are expressed as mean ± standard deviation (SD) or medians (P_25_, P_75_). Statistical analyses were performed using one-way analysis of variance (ANOVA) followed by the Least Significant Difference (LSD) test using SPSS version 20.0 (IBM Corporation, Armonk, NY, USA). Lung injury and Flameng scores were analyzed using Kruskal–Wallis one-way ANOVA. Repeated data were assessed by repeated measures ANOVA. Differences with *P* < 0.05 were considered to be statistically significant.

## Results

### H_2_ ameliorated oxidative stress and the inflammatory response

After 4 h cold ischemia, the W/D ratio in the control group (4.55 ± 0.54) was remarkably higher than the sham group (3.86 ± 0.38) (*P* < 0.05), whereas W/D ratio in groups O (4.25 ± 0.44) and H (4.13 ± 0.71) were not statistically significant compared with the control group. MPO activity in the control group (3.18 ± 1.34 U/g) was remarkably higher than in the sham group (0.35 ± 0.22 U/g), and that in H group (0.91 ± 0.29 U/g) exhibited marked reduction compared with O group (1.71 ± 0.75 U/g) (*P* < 0.05). The tendencies of IL-6, TNF-α and MDA were similar to MPO, which were in contrast to the anti-inflammatory factor IL-10 and the antioxidant indicator SOD (Table 1).

**Table 1:** W/D ratio, inflammatory and oxidative stress indices (mean ± SD, *n* = 10)

	W/D ratio	IL-6 (pg/mL)	TNF-α (pg/mL)	IL-10 (pg/mL)	MPO (U/g)	SOD (U/mg)	MDA (nmol/mg)
Sham group	3.86 ± 0.38	3449 ± 686	131 ± 52	956 ± 78	0.35 ± 0.22	82 ± 6	0.66 ± 0.15
Control group	4.55 ± 0.54^*^	7188 ± 762^*^	406 ± 85^*^	503 ± 82^*^	3.18 ± 1.34^*^	52 ± 8^*^	2.21 ± 0.85^*^
O group	4.25 ± 0.44	6076 ± 835^*#^	309 ± 80^*#^	635 ± 111^*#^	1.71 ± 0.75^*#^	63 ± 9^*#^	1.68 ± 0.63^*#^
H group	4.13 ± 0.71	4583 ± 897^*#§^	212 ± 81^*#§^	811 ± 96^*#§^	0.91 ± 0.29^#§^	73 ± 12^*#§^	1.18 ± 0.25^*#§^

*IL* Interleukin, *MDA* Malondialdehyde, *MPO* Myeloperoxidase, *SOD* Superoxide dismutase, *TNF-α* Tumor necrosis factor-α, *W/D ratio* Wet/dry weight ratio

^*^P < 0.05 vs. Sham group; ^#^*P* < 0.05 vs. Control group; ^§^*P* < 0.05 vs. O group

### H_2_ alleviated pathological injury in donor lungs

**Fig. 1:**
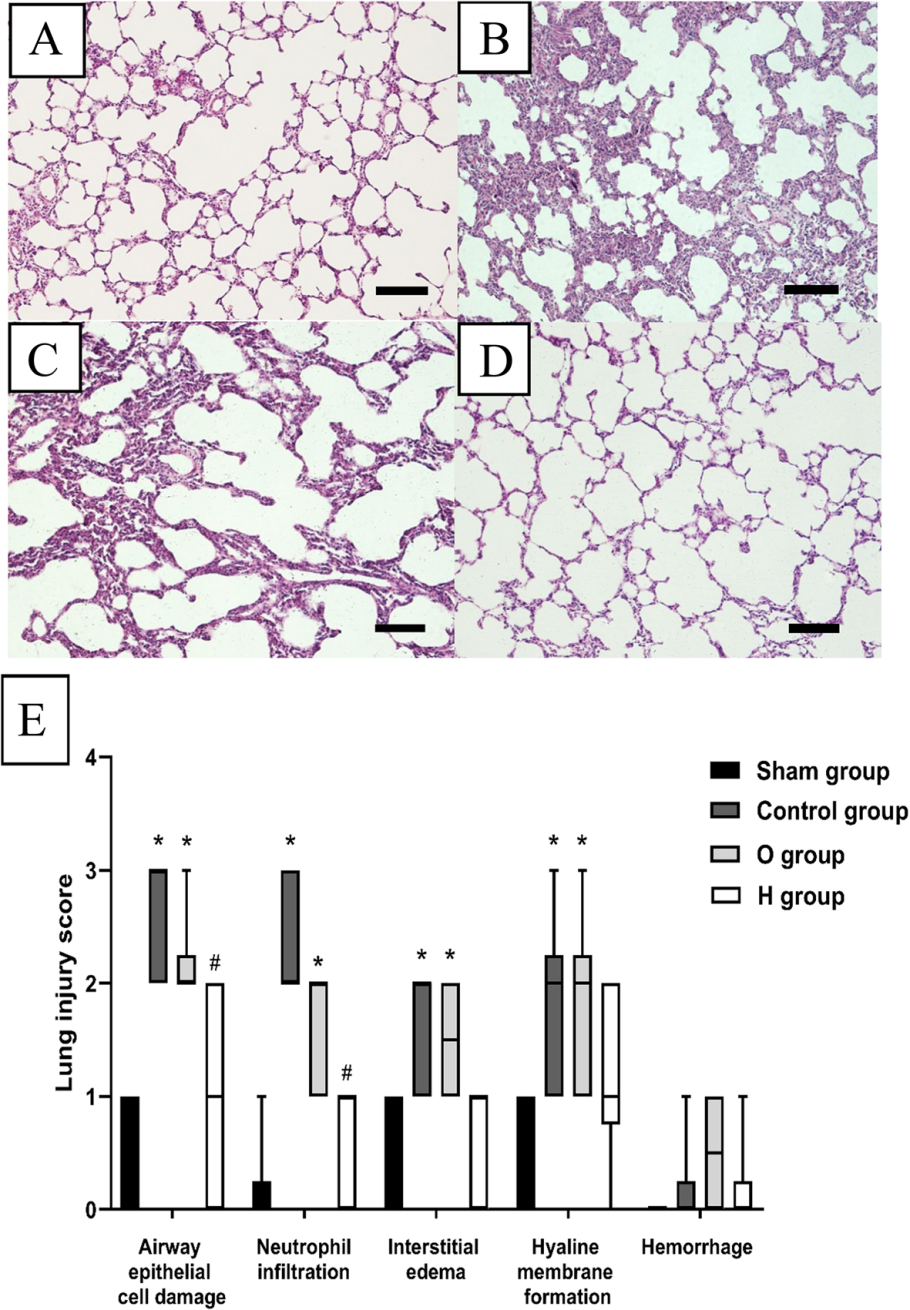
Histologic analysis of lung tissues (*n* = 6, 10 ×). **A** Sham group; **B** Control group; **C** O group; **D** H group; **E** Lung injury score (LIS) for each criterion. Scale bar: 500 μm. The LISs in the Control group and O group were higher than the Sham group (*P* < 0.05). The LISs of airway epithelial cell damage and neutrophil infiltration in H group were lower than the Control group (*P* < 0.05), however, LIS of hemorrhage was not statistically significant among these groups. ^*^P < 0.05 vs. Sham group; ^#^*P* < 0.05 vs. Control group

Microscopic analysis revealed normal lung parenchyma in the sham group (Fig. 1A). However, moderate airway epithelial cell damage, neutrophil infiltration, mild interstitial edema and hyaline membrane formation were evident in the control group (Fig. 1B). Fewer damage changes were observed in O and H groups, and tissue injury was milder following H_2_ treatment (Fig. 1C and D). Specifically, the LIS for airway epithelial cell damage in the control group (3 [2–3]) exhibited a meaningful increase compared with the sham group (0.5 [0–1]) (*P* < 0.05). The LIS for O group (2 [1–3]) was diminished compared with the control group, and that for H group (1 [0–2]) was also lower than O group (*P* < 0.05). Simultaneously, the LISs of other criteria demonstrated tendencies analogous to airway epithelial cell damage, except for intra-alveolar hemorrhage that demonstrated no statistical significance in all groups (Fig. 1E).

### H_2_ suppressed cell apoptosis in donor lungs

**Fig. 2:**
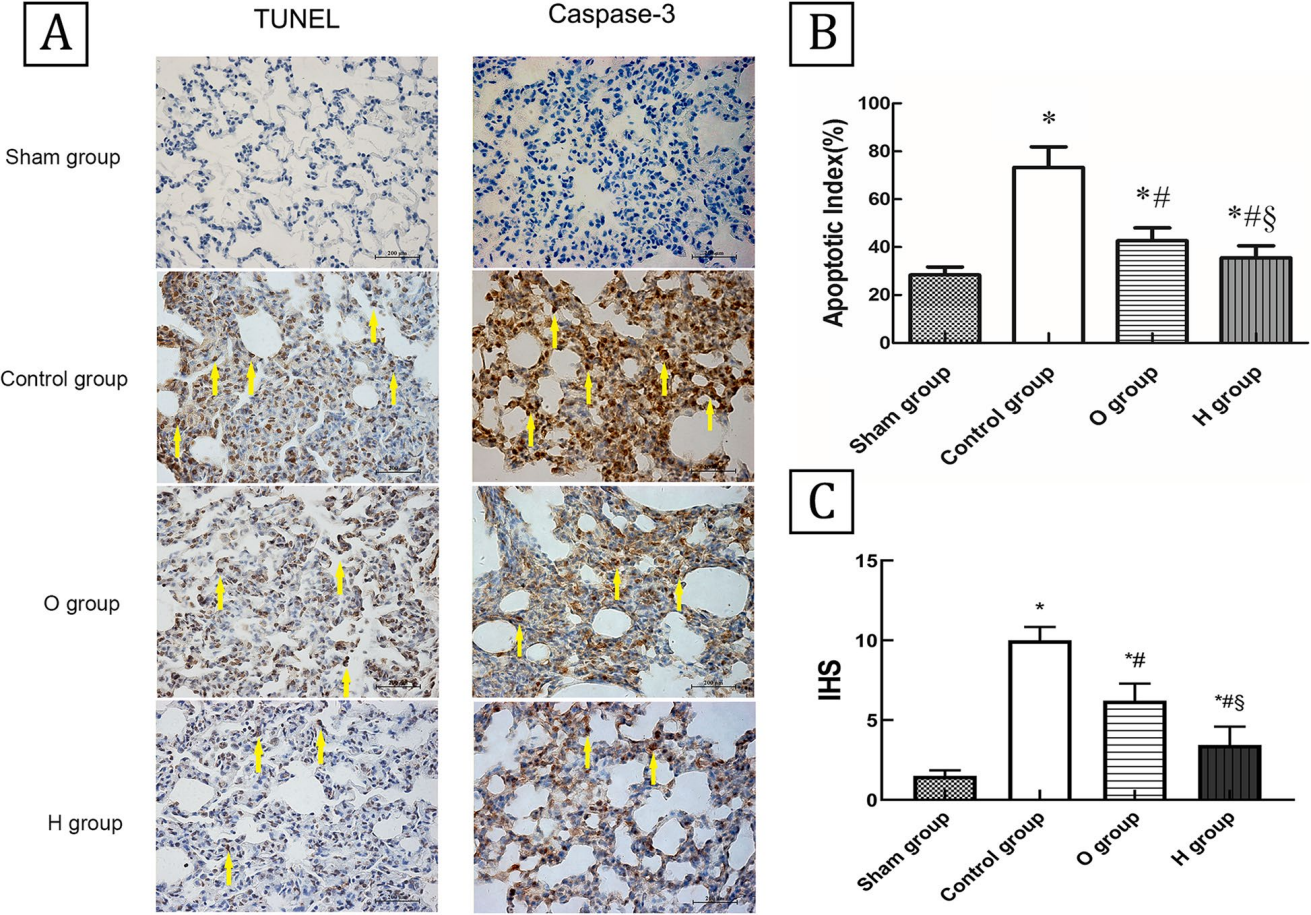
Hydrogen alleviated alveolar epithelial cell apoptosis and suppressed the expression of caspase-3 (*n* = 6, 40 ×). **A** Representative staining of apoptotic cells with TUNEL-positive (Scale bar: 200 μm) and caspase-3 positive (Scale bar: 100 μm) in four groups. **B** The apoptotic index of TUNEL. The number of apoptotic cells was higher in Control group than Sham group; O group had fewer apoptotic cells than Control group, and H group exhibited more slighter apoptosis comparing with O group (*P* < 0.05). **C** The HIS of caspase-3. The Control group had a higher IHS than the Sham group; the IHS of O group was decreased than Control group, and the IHS of H group was lower than O group (*P* < 0.05). TUNEL: terminal deoxynucleotidyl transferase-mediated dUTP nick end labeling. IHS: Immunohistochemical score. ^*^P < 0.05 vs. Sham group; ^#^*P* < 0.05 vs. Control group; ^§^*P* < 0.05 vs. O group

Representative staining of apoptotic cells with TUNEL-positive and caspase-3 positive were shown in Fig. 2A. Compared with the sham group (28.4 ± 3.3), abundant TUNEL-positive cells were observed in the control group (73.3 ± 8.5) (*P* < 0.05). Positive cell counts in O group (42.7 ± 5.3) exhibited significant improvement compared with the control group. Additionally, the result in H group (35.5 ± 5.0) was lower than that for O group (*P* < 0.05) (Fig. 2B). Similarly, caspase-3 expression evaluated according to IHS was higher in the control group (10.0 ± 0.8) than the sham group (1.7 ± 0.5), the IHS in O group (6.2 ± 1.1) was lower than the control group, while the value in H group (3.2 ± 1.3) was lower than in O group (*P* < 0.05) (Fig. 2C).

### H_2_ enhanced ATP biosynthesis and energy metabolism and improved antioxidant activity in mitochondria

Compared with the control group (2.79 ± 0.51 nmol/mg prot), ATP production was significantly increased in the sham group (5.79 ± 0.58 nmol/mg prot) and in O group (3.92 ± 0.25 nmol/mg prot) (*P* < 0.05). Moreover, H group (5.18 ± 0.61 nmol/mg prot) exhibited greater ATP content than O group (*P* < 0.05). Glucose levels in lung tissue were lower in the control group (0.68 ± 0.13 μmol/mg prot) than in the sham group (1.67 ± 0.33 μmol/mg prot) (*P* < 0.05) but were higher in group O (1.00 ± 0.16 μmol/mg prot) compared to the control group (*P* < 0.05). Glucose levels in H group (1.20 ± 0.18 μmol/mg prot) were remarkably higher than in group O (*P* < 0.05). However, levels of lactic acid and pyruvic acid were contrary to glucose among these groups.

In the aspect of lipid peroxidation, mMDA level in the control group (4.87 ± 0.63 nmol/mg prot) was higher than in the sham group (2.07 ± 0.37 nmol/mg prot) (*P* < 0.05), while it was lower in H group (2.80 ± 0.88 nmol/mg prot) than that in group O (3.45 ± 0.76 nmol/mg prot) (*P* < 0.05). mGSH level in the control group (3.74 ± 0.46 nmol/mg prot) was significantly lower than in the sham group (7.69 ± 0.64 nmol/mg prot), while O group (5.13 ± 0.68 nmol/mg prot) exhibited an increase compared with the control group (*P* < 0.05), and mGSH level in H group (5.99 ± 0.82 nmol/mg prot) was higher than in O group (*P* < 0.05). mGSSG level exhibited a tendency reverse to that of mGSH (*P* < 0.05) (Table 2).

**Table 2:** The indexes of energy metabolism and lipid peroxidation (mean ± SD, *n* = 10)

	Sham group	Control group	O group	H group
***Energy metabolism***				
ATP *(nmol/mg prot)*	5.79 ± 0.58	2.79 ± 0.51^*^	3.92 ± 0.25^*#^	5.18 ± 0.61^*#§^
Glucose *(μmol/mg prot)*	1.67 ± 0.33	0.68 ± 0.13^*^	1.00 ± 0.16^*#^	1.20 ± 0.18^*#§^
Lactic acid *(mmol/g prot)*	0.25 ± 0.05	0.43 ± 0.08^*^	0.36 ± 0.04^*#^	0.30 ± 0.04^*#§^
Pyruvic acid *(μmol/mg prot)*	0.17 ± 0.04	0.35 ± 0.06^*^	0.30 ± 0.03^*#^	0.22 ± 0.06^*#§^
***Lipid peroxidation***				
mMDA *(nmol/mg prot)*	2.07 ± 0.37	4.87 ± 0.63^*^	3.45 ± 0.76^*#^	2.80 ± 0.88^*#§^
mGSH *(nmol/mg prot)*	7.69 ± 0.64	3.74 ± 0.46^*^	5.13 ± 0.68^*#^	5.99 ± 0.82^*#§^
mGSSG *(nmol/mg prot)*	0.36 ± 0.10	0.70 ± 0.11^*^	0.54 ± 0.07^*#^	0.45 ± 0.08^*#§^

*ATP* Adenosine triphosphate, *mMDA* mitochondrial malondialdehyde, *mGSH* mitochondrial glutathione, *mGSSG* mitochondrial glutathione disulfide

^*^P < 0.05 vs. Sham group; ^#^*P* < 0.05 vs. Control group; ^§^*P* < 0.05 vs. O group

### H_2_ protected mitochondrial structure

**Fig. 3:**
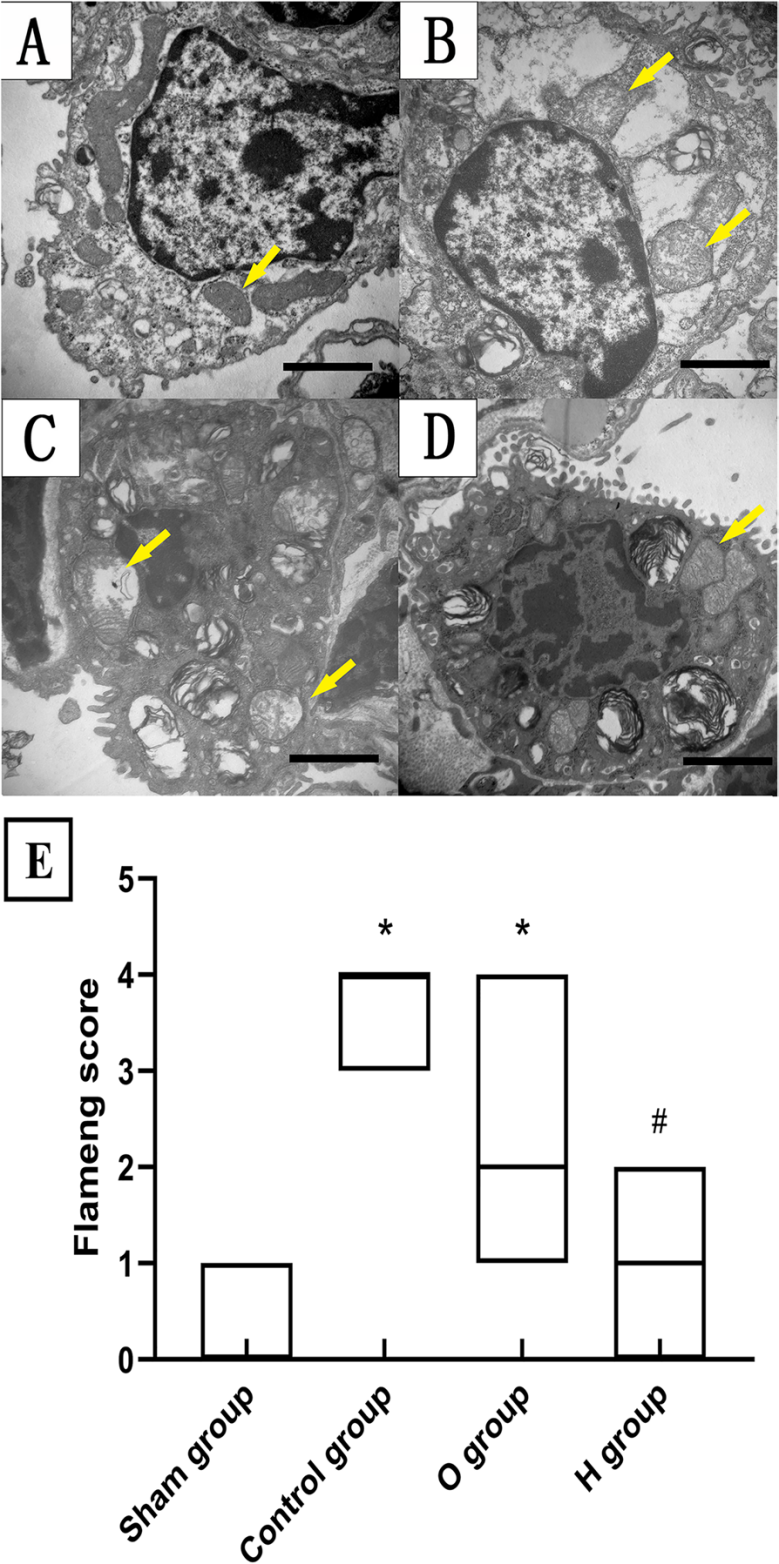
Hydrogen protected morphological characteristics of mitochondria under transmission electron microscopy (*n* = 7, 20,000 ×). **A** Sham group, the mitochondria of alveolar type II epithelial cells had a normal structure and intact particles; **B** Control group, the mitochondria had broken cristae and ruptured mitochondrial; **C** O group, the mitochondria had swollen and partly ruptured cristae, the injury was lighter than the Control group; **D** H group, the mitochondria mildly swelled and the injury was lighter than the O group but heavier than the Sham group. Yellow arrows indicate the mitochondria; **E** Flameng score of mitochondrial. Scale bar: 2 μm. ^*^P < 0.05 vs. Sham group; ^#^*P* < 0.05 vs. Control group

Mitochondrial structure in the sham group remained essentially normal (Fig. 3A), while it was severely damaged in the control group, with the presence of many lipid droplets and vacuoles, serious mitochondrial edema, matrix concentration, mitochondrial membrane, and cristae rupture or deficiency (Fig. 3B). Mitochondrial swelling and partial crest rupture in O group were mitigated, in contrast to the control group, and also clearly ameliorated in H group compared with O group (Fig. 3C and D). Similarly, Flameng scores in the control group (4 [3–4]) and O group (2 [2–3]) were both higher than in the sham group (0 [0–1]) (*P* < 0.05), and scores for group H (1 [0–2]) were lower than the control group (*P* < 0.05). However, the scores between groups O and H had no statistical differences (*P* > 0.05) (Fig. 3E).

### H_2_ increased MMP and inhibited mitochondrial swelling

**Fig. 4:**
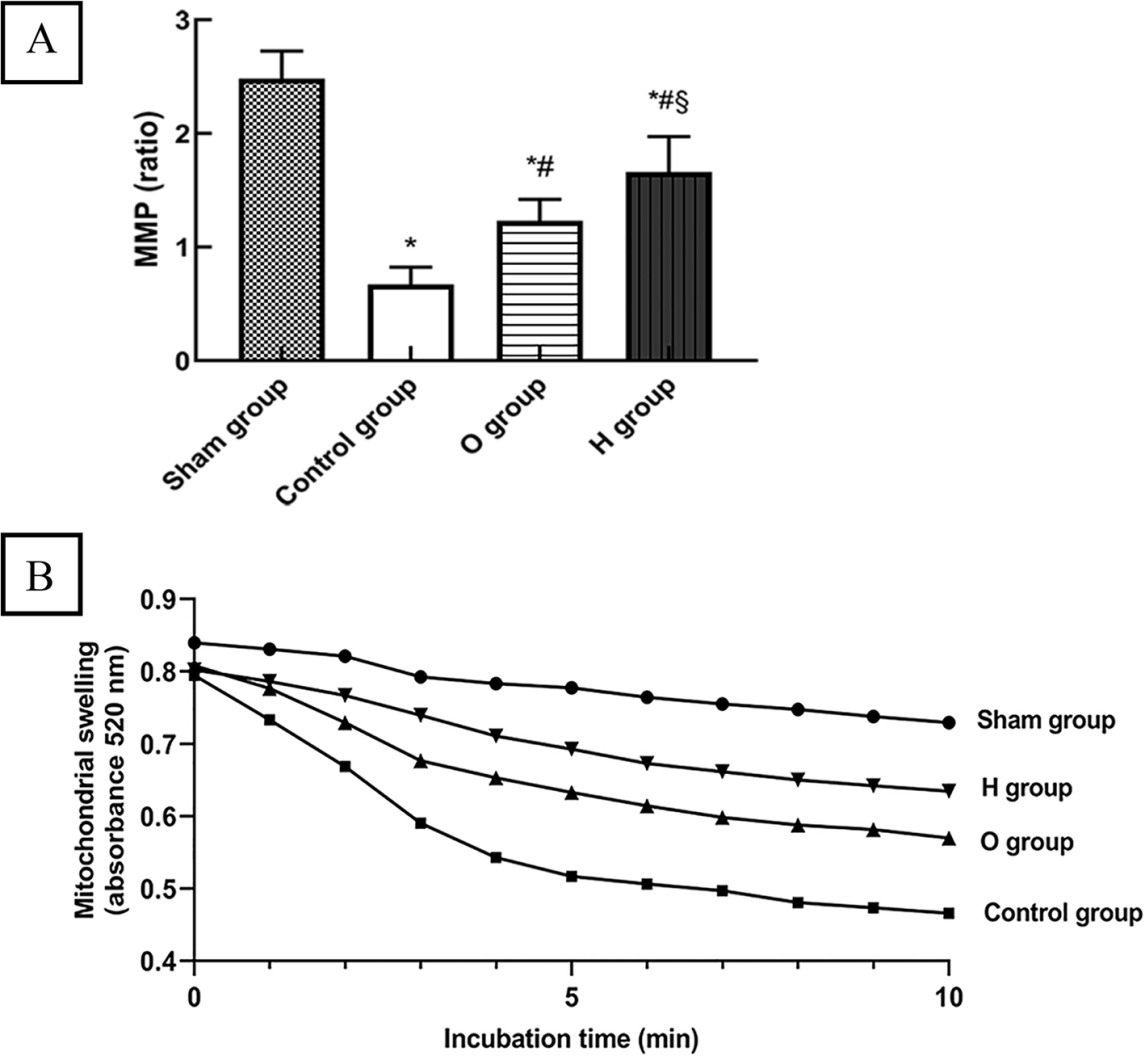
Hydrogen increased MMP and inhibited mitochondrial swelling (*n* = 6). (A) The MMP was calculated by the ratio of red fluorescent aggregates to green fluorescent monomers. The Control group had a lower MMP than the Sham group; the MMP in O group was increased significantly than Control group, and H group showed a higher MMP than O group (*P* < 0.05). (B) Mitochondrial swelling was assayed at 520 nm absorbance. The decrease of absorbance was negative correlated with the degree of mitochondrial swelling. Mitochondria from the Sham group was basically normal. Conversely, large‑amplitude swelling was observed in Control mitochondria; mitochondrial edema was alleviated in H group compared with the O group. MMP: Mitochondrial membrane potential. ^*^P < 0.05 vs. Sham group; ^#^*P* < 0.05 vs. Control group; ^§^*P* < 0.05 vs. O group

MMP in the control group (0.67 ± 0.14) was lower than in the sham group (2.48 ± 0.23) (*P* < 0.05). Group O (1.23 ± 0.18) exhibited a higher ratio than the control group (*P* < 0.05), while the value in H group (1.66 ± 0.30) was higher than that in O group (*P* < 0.05) (Fig. 4A).

Mitochondrial swelling is quantitatively analyzed by the absorbance value at 520 nm. Mitochondria from the sham group did not exhibit mitochondrial permeability transition pore (mPTP). Conversely, large‑amplitude swelling was observed in control mitochondria compared to the sham group. Simultaneously, mitochondrial edema was alleviated in H group compared with O group, perhaps suggesting that H_2_ treatment prevented-or at least mitigated-mitochondrial swelling (Fig. 4B).

### H_2_ activated Nrf2 and HO-1 expression

**Fig. 5:**
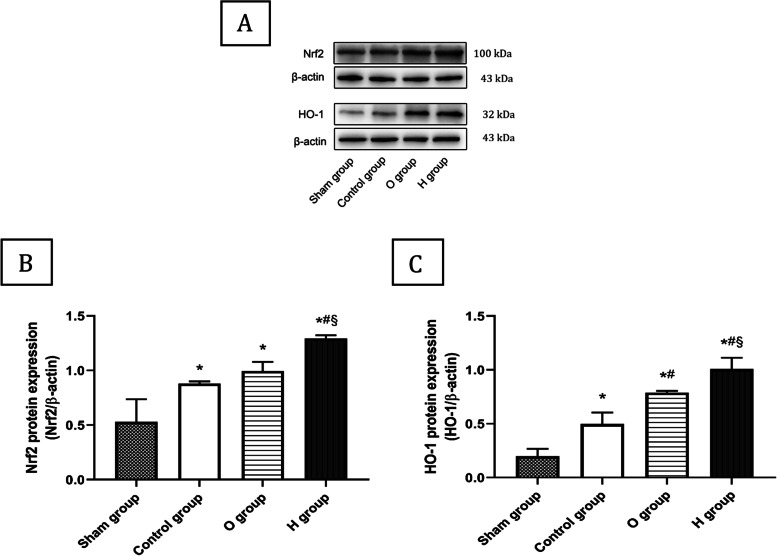
Hydrogen promoted the proteins expression of Nrf2 and HO-1 (*n* = 3). The relative expression levels of Nrf2 or HO-1 presented as the ratio of band density of Nrf2 or HO-1 to β-actin. Data were presented as mean ± SD. The Nrf2 and HO-1 protein levels were higher in the Control group than the Sham group; the content of HO-1 in O group was also increased than the Control group (*P* < 0.05). Moreover, H group obviously increased the Nrf2 and HO-1 contents when comparing with the O group (*P* < 0.05). Nrf2, nuclear factor erythroid 2-related factor 2; HO-1, heme oxygenase-1. ^*^P < 0.05 vs. Sham group; ^#^*P* < 0.05 vs. Control group; ^§^*P* < 0.05 vs. O group

As shown in Fig. 5, the protein expression levels of Nrf2 and HO-1 in the control group were significantly increased compared with those in the sham group, and the HO-1 protein level were also higher in O group than in the control group (*P* < 0.05). Additionally, comparing with the O group, H_2_ remarkably promoted the Nrf2 and HO-1 protein levels, suggesting that H_2_ was a potent activator of the Nrf2 pathway in response to lung injury during CIP (*P* < 0.05). The original blots/gels are presented in Fig. S1.

## Discussion

In current study, H_2_ treatment decreased the levels of MPO, MDA, IL-6 and TNF-α, increased the levels of IL-10 and SOD, alleviated donor lung pathological injury, inhibited caspase-3 expression and cell apoptosis, decreased the contents of mMDA and mGSSG while augmenting mGSH level, suppressed mitochondrial swelling, facilitated MMP and energy metabolism, as well as up-regulated the expression of Nrf2 and HO-1.

Increasing evidence has suggested that H_2_ exerts various biological effects, including anti-oxidative stress, anti-inflammation, anti-apoptosis and anti-pyroptosis [19, 20], while regulating endoplasmic reticulum stress, and mitochondrial and immunological function [21]. Additionally, H_2_ also modulates some intracellular signaling pathways, such as Nrf2/HO-1, NF-κB, MAPK, aquaporin-1 and aquaporin-5 [22]. Noda et al*.* suggested that H_2_ preconditioning attenuated pro-inflammatory responses, decreased lactic acid production, downregulated hypoxia inducible factor-1 level, enhanced the activation of mitochondrial complex I, II, and IV, and promoted mitochondrial biogenesis in an EVLP model [23]. Chen et al*.* verified that H_2_-rich saline protected against organ injury from sepsis via down-regulation of endoplasmic reticulum stress-related markers and pro-inflammatory factors, activation of autophagy and inactivation of the endoplasmic reticulum stress pathway [24]. In the present study, H_2_ inflation regulated mitochondrial structure and function in donor lung by inhibiting oxidative stress, inflammation, and cell apoptosis, promoting MMP and mitochondrial energy metabolism, contributing to improvement in donor lung quality, which further verified the protective effects of H_2_. The technique of inflation using H_2_ in donor lung may lay a foundation for the application of H_2_ in clinical practice.

Inflammatory response and oxidative stress play important roles in lung injury during CIP. Oxidative stress and mitochondrial dysfunction are common features in many inflammatory diseases. Mitochondrial ROS induced by mitochondrial dysfunction has been shown to contribute to local inflammatory pathologies induced by lipopolysaccharide from Gram-negative bacteria [25]. Thus, inflammation and oxidative stress are potential therapeutic targets to alleviate tissue injury. Terasaki et al*.* reported that molecular H_2_ mitigated acute lung injury through increasing GSH, lowering MDA and 4-hydroxy-2-nonenal production, inducing the repression of oxidative stress and inflammation in the lung tissue and airway wall [26]. Zhang et al*.* reported that donor lung inflation using 3% H_2_ during the warm ischemia phase decreased pro-inflammatory factors levels and caspase-3 and nuclear NF-κB protein expression, inhibited the indices of oxidative stress, contributing to the improvement of post-transplantation pulmonary function after cardiac death [27]. Similarly, our results demonstrated that H_2_ down-regulated levels of IL-6, TNF-α, MPO and MDA, enhanced the expression of IL-10 and SOD, ameliorated the severity of lung injury in grafts. These were also evidenced by histological examination, indicating that H_2_ could ultimately mitigate donor lung injury by anti-inflammatory and antioxidant effects.

Mitochondrial membrane lipid peroxidation induces mitochondrial dysfunction in the presence of ROS; as a critical antioxidant, mGSH can be oxidized to mGSSG, which does require transport from the mitochondria to cytoplasm and is catalyzed by several enzymes in the inner mitochondrial membrane, finally generating mMDA. Recent evidence has demonstrated that H_2_ regulates mitochondrial dysfunction by withstanding lipid peroxidation. For example, Sakai et al*.* suggested that the application of supersaturated H_2_ increased GSH levels and inhibited membrane lipid peroxidation in a cell senescence model [28]. Liu et al*.* reported that H_2_‑rich saline significantly attenuated mGSSG and mMDA levels and augmented mGSH expression, markedly preventing the elevation of mitochondrial lipid peroxidation in bile duct ligation‑induced liver injury [29]. Consistent with above research, our data demonstrated that mMDA and mGSSG levels decreased and mGSH level increased following H_2_ inflation, indicating that H_2_ could suppress mitochondrial membrane lipid peroxidation and enhance the antioxidant capacity of mitochondria.

Mitochondria are the major source and target of excessive ROS. Nonetheless, ROS stimulates the consistent opening of mPTP, [30] directly causing mitochondrial membrane depolarization, the collapse of MMP, augmentation of osmotic pressure of matrix proteins, the release of cytochrome *c*, and irreversible cell apoptosis [31]. Additionally, intracellular calcium overload promotes anaerobic glycolysis, resulting in lower glucose levels and increased lactic acid and pyruvic acid levels by activation of AMP-activated protein kinase [32]. All these changes ultimately lead to disruption of energy metabolism, mitochondrial swelling, and apoptosis. In the present research, mitochondrial damage was severe in the control group, and mainly manifested as lower MMP, ATP and glucose synthesis, significant mitochondrial swelling, and higher levels of pyruvic acid and lactic acid. Despite the similar oxygen concentration administered to groups O and H, H_2_ inflation yielded better outcomes than O_2_ alone, suggesting that H_2_ inflation safeguarded mitochondrial structure and function by decreasing metabolic rate or energy demand during CIP. These data were in accordance with previous outcomes of sepsis-induced acute lung injury [33].

Accumulating evidence indicates that H_2_ participates in the regulation of Nrf2 signaling pathway, which is closely associated with oxidative stress. According to Sun’s study, 67% H_2_ inhalation alleviated LPS-induced acute lung injury via improving lung cell apoptosis, inflammatory response, and activating Nrf2 and subsequently inhibiting NF-κB pathway [34]. In an H_2_O_2_-induced neuroblastoma cell death model, Murakami et al*.* reported that pretreatment with 50% H_2_ up-regulated mRNA expression of Nrf2 and HO-1, augmented MMP and ATP production, enhanced mitochondrial resistance to oxidative stress and consequently induced mitohormesis [35]. Zhang et al*.* also reported that 2% H_2_ inhalation raised the mitochondrial respiratory control ratio, MMP and ATP synthesis in a mouse model of sepsis-induced cardiomyocyte injury, and knockdown of the *Nrf2* gene weakened these effects [36]. The current study also revealed that H_2_ increased Nrf2 and HO-1 expression, decreased damage caused by oxidative stress, and protected against mitochondrial injury, as evidenced by decreased MDA and MPO expression and increased SOD levels. Thus, all of these outcomes suggest that H_2_ inflation protects mitochondrial function from oxidative stress injury, which may be related to the Nrf2 signaling pathway.

There were several limitations to the present study. Firstly, except for donor lung, recipients should be treated with H_2_ to verify the impact of H_2_ on lung reperfusion injury. Secondly, indicators of pulmonary ventilation function, such as lung compliance and oxygenation capacity, were not measured. Thirdly, the Nrf2/HO-1 pathway was not verified by applying relevant inhibitors, and some interventions need to be conducted in future research. Finally, only one H_2_ concentration was applied in this study. Whether the protective effect of H_2_ is closely related to the concentration should be further analyzed. As the further study in our team, 66.7% H_2_ (produced by a hydroxide atomizer) has proven to be lung protective (data unpublished), which may be applied in clinical application of lung transplant.

## Conclusions

In summary, this study demonstrated that donor lungs experienced compromised mitochondrial dysfunction during CIP and exhibited significant injury. Lung inflation using H_2_ could improve donor lung quality through mitigating disorder of mitochondrial structure and function, which may be achieved via activation of the Nrf2/HO-1 pathway.

## Supplementary Information

**Additional file 1: **Original images of Western blot.

## Data Availability

The datasets generated in this study are available from the corresponding author upon reasonable request.

## Abbreviations

LTx: Lung transplantation
ROS: Reactive oxygen species
H2: Hydrogen
EVLP: Ex vivo lung perfusion
O2: Oxygen
IRI: Ischemia-reperfusion injury
CIP: Cold ischemia phase
MMP: Mitochondrial membrane potential
Nrf2: Nuclear factor erythroid 2-related factor 2
HO-1: Heme oxygenase-1
SOD: Superoxide dismutase
LPD: Low-potassium dextran
W/D: Wet-dry weight ratio
MDA: Malondialdehyde
MPO: Myeloperoxidase
IL: Interleukin
TNF-α: Tumor necrosis factor-alpha
LIS: Lung injury score
TUNEL: Terminal deoxynucleotidyl transferase-mediated dUTP nick end labeling
AI: Apoptotic index
HIS: Immunohistochemical score
ATP: Adenosine triphosphate
mGSH: Mitochondrial glutathione
mGSSG: Mitochondrial glutathione disulfide
mPTP: Mitochondrial permeability transition pore
