# Supplementary material for: Inflation using hydrogen improves donor lung quality by regulating mitochondrial function during cold ischemia phase
Source: BMC Pulm Med. 2023 Jun 17;23:213. doi: 10.1186/s12890-023-02504-6 (PMC10276452; doi:10.1186/s12890-023-02504-6)
Supplement: Supplementary file 1 — Additional file 1: Original images of Western blot. [file 12890_2023_2504_MOESM1_ESM.pdf]

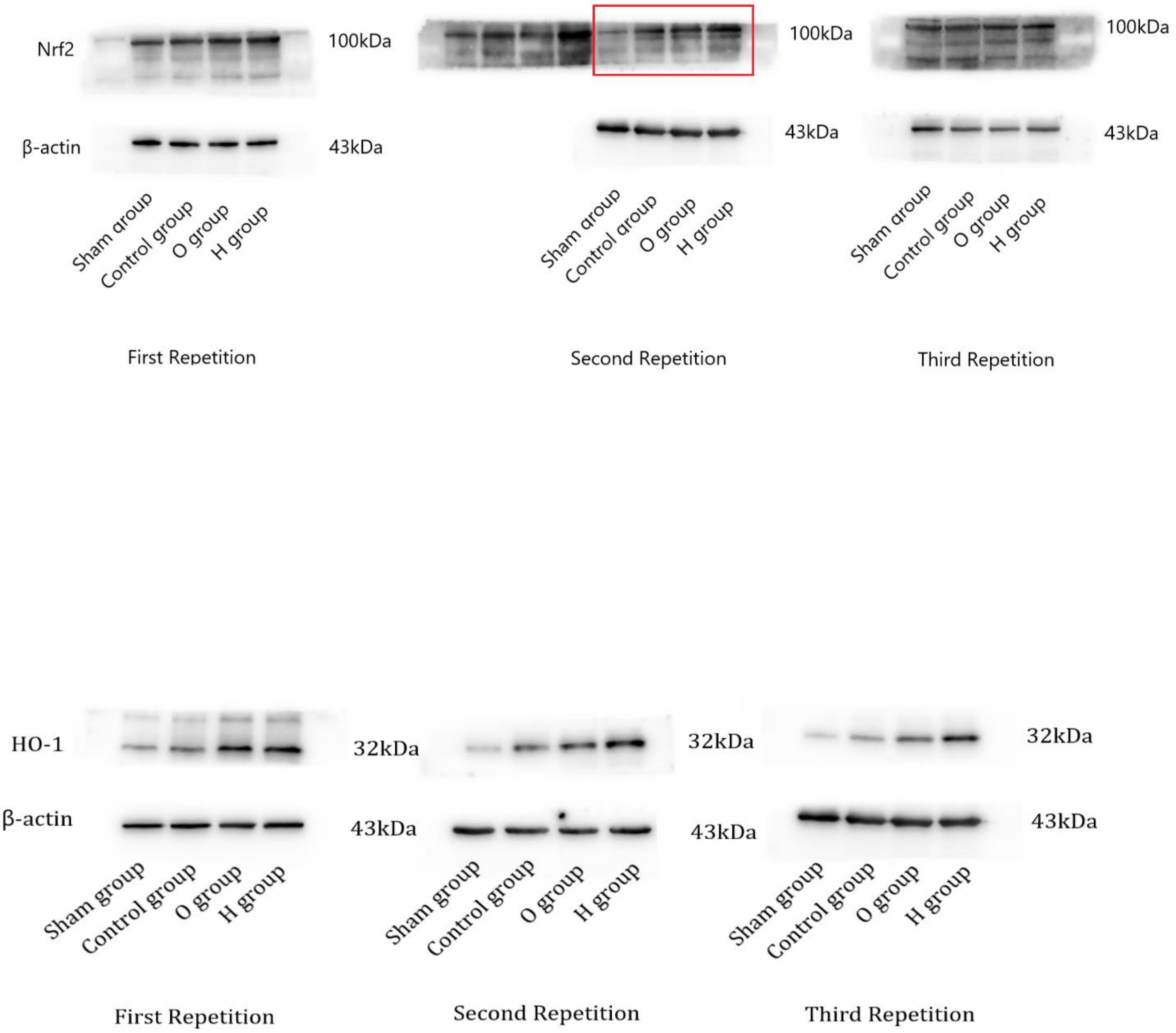

Fig S1. Original Western blot images of Fig 5A. The blots were cut prior to hybridization with antibodies. The images show all blots and replicates of Nrf2, HO-1, β-actin. Nrf2, nuclear factor erythroid 2-related factor 2; HO-1, heme oxygenase-1.
